# Primary Cilia: The New Face of Craniofacial Research

**DOI:** 10.3390/biom12121724

**Published:** 2022-11-22

**Authors:** Emily R. Moore

**Affiliations:** Harvard School of Dental Medicine, Department of Developmental Biology, 188 Longwood Avenue, Boston, MA 02115, USA; emily_moore@hsdm.harvard.edu

**Keywords:** primary cilia, ciliopathies, cleft palate, cleft lip, dental pulp stem cells, odontoblasts, neurons, Hh signaling, Wnt signaling, calcium signaling

## Abstract

The primary cilium is a solitary, sensory organelle that extends from the surface of nearly every vertebrate cell, including craniofacial cells. This organelle converts chemical and physical external stimuli into intracellular signaling cascades and mediates several well-known signaling pathways simultaneously. Thus, the primary cilium is considered a cellular signaling nexus and amplifier. Primary cilia dysfunction directly results in a collection of diseases and syndromes that typically affect multiple organ systems, including the face and teeth. Despite this direct connection, primary cilia are largely unexplored in craniofacial research. In this review, I briefly summarize craniofacial abnormalities tied to the primary cilium and examine the existing information on primary cilia in craniofacial development and repair. I close with a discussion on preliminary studies that motivate future areas of exploration that are further supported by studies performed in long bone and kidney cells.

## 1. Introduction

**Figure 1:**
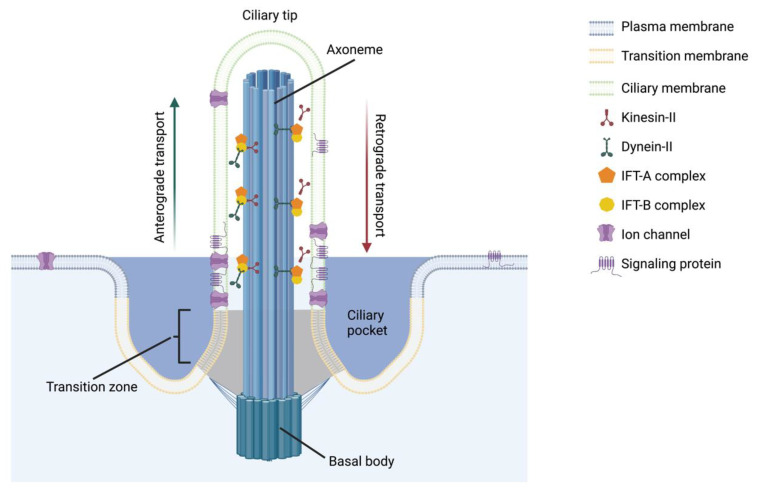
Primary cilium structure and intraflagellar transport (IFT) components. The primary cilium is composed of an anchoring basal body and an axoneme that extends from the cell surface. The axoneme is encapsulated by a ciliary membrane that is distinct from but continuous with the plasma membrane. Signaling proteins, such as ion channels and receptors, are especially concentrated near the axoneme base to facilitate signaling amplification. The axoneme structure is maintained by IFT and its components including kinesin-II, dynein-II, IFT-A, and IFT-B. This figure was created with BioRender.com (accessed on 10 November 2022).

The primary cilium is a solitary sensory organelle that projects from the cell surface of most vertebrate cells, including bone and dental cells. It operates as both a chemo- and mechanosensor to transduce external stimuli into intracellular signaling cascades. This nearly ubiquitous structure is comprised of an axoneme that extends from a basal body, which helps anchor the axoneme to the cell (Figure 1). The point at which the proximal portion of the primary cilium meets the basal body is termed the *transition zone* because it functions as a gate for proteins entering and exiting the ciliary domain. The axoneme contains nine microtubule doublets whose assembly and maintenance are dependent on intraflagellar transport (IFT). Motor proteins associate with complexes containing IFT proteins (IFT-A or B) and travel along the microtubule doublets, delivering or removing cargo necessary for axoneme maintenance. Kinesin-II is thought to attach to IFT-B to carry this complex, as well as IFT-A and inactive dynein-II, to the ciliary tip in *anterograde transport* [1,2]. Near the ciliary tip, kinesin-II will dissociate and dynein-II becomes activated. Dynein-II is believed to bind with IFT-A and returns the IFT complexes and any other necessary cargo to the axoneme base, a process referred to as *retrograde transport* [1,2]. The axoneme can adjust its length via IFT to modify sensitivity of the primary cilium and subsequent cell behavior, qualifying it as a transient structure. The axoneme is surrounded by a ciliary membrane that is distinct from the cell membrane. Many illustrations of the primary cilium depict a smooth, continuous fusion of the ciliary and plasma membranes, but the primary cilium is slightly recessed such that a “pocket” is formed. The ciliary pocket has yet to be fully characterized but is believed to be important for vesicle trafficking and interaction with the actin cytoskeleton. 

Proteins associated with various cell signaling pathways are present in the ciliary domain through a process tightly regulated by the transition zone. Some of these proteins are unique to the primary cilium and many are highly concentrated in the ciliary domain, especially near the axoneme base, compared to the rest of the cell. This focused assortment of cell signaling components enables the primary cilium to mediate and amplify a variety of signaling pathways important for bone and dental cell behavior. Ciliary signal transduction can be distinct from signaling orchestrated by the cell membrane, but in some instances it is complementary or additive. Based on these characteristics, the primary cilium is a cellular antenna that operates as a signaling nexus to direct cell behavior. The cilium is so critical that alterations in IFT machinery or transition zone utility which disrupt primary ciliary function cause an assortment of human diseases called *ciliopathies*. Ciliopathies typically affect multiple organs simultaneously, including the craniofacial tissues and dentition.

In this review, I will first summarize the most prevalent ciliopathies affecting craniofacial structures. Next, I will discuss existing research that demonstrates the importance of primary cilia in dental cell behavior and development of craniofacial tissues. I will close with an exploration of primary cilia characteristics observed in other tissues that are worth further investigation in dental cells.

## 2. Craniofacial Defects Associated with Ciliopathies

Ciliopathies are diseases that directly result from alterations in the primary cilium itself or components associated with the ciliary complex that render the primary cilium dysfunctional. These conditions typically affect multiple organ systems and approximately 30% of ciliopathies involve craniofacial and dental defects including cleft lip and palate, dental hypoplasia, midfacial hypoplasia, craniosynostosis, retrognathia/micrognathia, and irregularities in the number, size, or anatomical location of teeth [3,4,5,6]. Certain conditions initially categorized as craniofacial diseases have since been recognized as official ciliopathies. Of note, Frontonasal Dysplasia (FND) and Cranioectodermal Dysplasia (CED), also known as Sensenbrenner syndrome, were proposed for consideration a decade ago [4]. CED has since been directly linked to mutations in intraflagellar transport protein 122 (IFT122), establishing its place in the list of ciliopathies [5,7,8] Another group developed a mouse model for FND by deleting a subunit of the kinesin-2 motor protein (KIF3A) from neural crest cells [9]. *Kif3a* was once a common target for primary cilia disruption because it is important for axoneme maintenance and subsequent ciliary signaling. These mice exhibit classic symptoms of FND such as cleft palate, cleft skull, bifid nasal septum, and agenesis of corpus callosum [9], providing strong evidence in support of classifying FND as a ciliopathy. 

Several diseases primarily impacting the craniofacial tissues have long been considered ciliopathies. The most prevalent or well-characterized are Joubert syndrome and its allelic counterpart Meckel syndrome, Oral–facial–digital syndrome, Ellis-van Creveld (EVC) syndrome, and Weyers acrofacial dysostosis. These syndromes are directly linked to mutations in components of the primary cilia complex, especially IFT proteins and proteins associated with trafficking in the ciliary pocket (see Figure 3) [5,7,10,11,12,13]. Depending on the mutation, primary cilia dysfunction is caused by compromised axoneme structure resulting from disrupted IFT, ciliary protein trafficking, or basal body stability. In some cases, primary cilia appear structurally normal but cannot sense, transduce, or respond to external stimuli. Joubert and Meckel syndrome patients can exhibit a variety of symptoms but typically experience brain abnormalities due to defective skull formation, kidney disease, and polydactyly. Oral–facial–digital syndrome patients have similar kidney and digit issues but this disease is mostly known for its accompanying collection of oral symptoms like cleft lip/tongue, growths on the tongue, missing or malformed teeth, and ectopic oral connective tissues. Patients with EVC syndrome have shortened limbs and cleft lip/palate due to mutations in the EVC/2 genes. Weyers acrofacial dysostosis also arises from mutations in these genes so it is not surprising that it shares many clinical features with EVC syndrome. However, these patients typically present with even more dental abnormalities including misshapen and missing teeth. Comprehensive lists of symptoms and ciliary genes associated with the aforementioned conditions, as well as craniofacial symptoms identified in classic ciliopathies, are detailed in previous reviews [4,5,7,12,14].

Increased awareness of the primary cilium’s involvement in these craniofacial diseases has sparked investigation of dental abnormalities in classic ciliopathies, such as Bardet–Biedl Syndrome (BBS), autosomal dominant polycystic kidney disease (ADPKD), and primary cilia dyskinesia (PCD). BBS is perhaps one of the most severe ciliopathies due to the wide range of organs affected, so it is not surprising that craniofacial tissues exhibit abnormalities. ADPKD patients develop fluid-filled cysts in the kidneys that often result in complete renal failure by the age of 60. One clinical report identified a dentigerous cyst in the lower jaw of a 42-year-old ADPKD patient [15]. The cyst and associated impacted tooth were removed and examination of the cystic lining by scanning electron microscopy revealed primary cilia emanated from epithelial cells into the cystic cavity. The authors speculated that ciliary defects associated with kidney cyst formation may also be related to the formation of this patient’s dental cyst, but more detailed examination in other ADPKD cases is required for a definitive link. PCD is characterized by a variety of chronic respiratory issues, as well as infertility in men. A small study of four adult PCD patients was conducted to determine whether dental defects were present. The phenotype was heterogenous but multiple patients exhibited enamel defects, bent and shortened tooth roots, and small or missing teeth [6]. 

More ciliopathies potentially have secondary clinical features in the dentition that remain undetected or poorly characterized simply because they are overshadowed by disastrous impairments in other parts of the body. One study determined that polycystin-1 (PC1), a ciliary protein associated with ADPKD, is expressed in the dental follicle cells that give rise to dentigerous cysts [16]. Furthermore, PC1 was detected in dentigerous cysts collected from human patients and the authors speculate that ADPKD patients may be at further risk for dentigerous cyst formation. It can also be difficult to distinguish whether primary cilium dysfunction is a direct or indirect cause of dental defects. In other words, dental abnormalities could arise from systemic changes rather than primary cilia dysfunction in the niche. Chronic kidney issues are known to negatively impact gum and tooth health independent of dental hygiene practices. Thus, it is possible the aforementioned ADPKD patient developed a dentigerous cyst over time as a result of kidney failure independent of primary cilia function in dental follicle cells. This remains an open question until mechanisms of cyst formation in different tissues are determined. The genetic determinants of ciliopathies are well characterized, therefore increasing our understanding of associated developmental defects in dental and craniofacial tissues can inform early detection and treatment, particularly in children. 

## 3. Primary Cilia in Dental Cells

**Figure 2:**
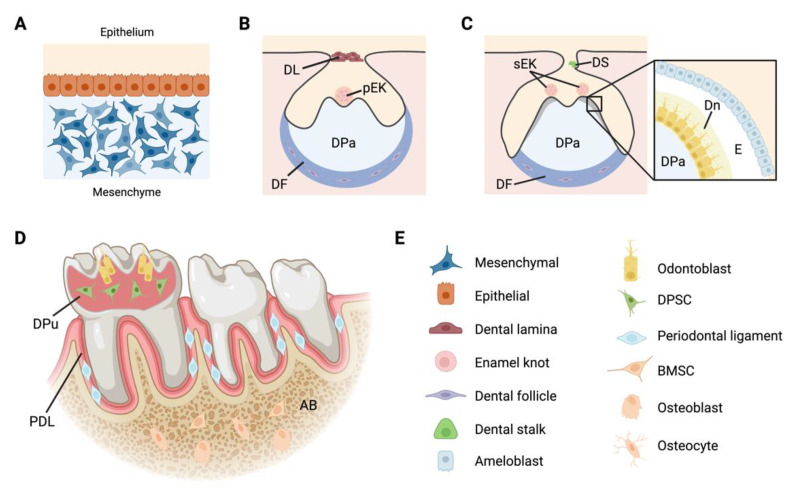
Dental cells known to contain primary cilia. Primary cilia are reported in cells that participate in tooth development (**A**–**C**) and make up the structures associated with fully formed teeth (**D**). (**A**) Cilia are detected as early as the initiation stage. (**B**) Cap stage: dental lamina (DL), primary enamel knot (pEK), dental papilla (DPa), dental follicle (DF). (**C**) Bell stage with magnified region where dentin (Dn) and enamel (**E**) are initially deposited: dental stalk (DS) and secondary enamel knot (sEK). (**D**) Fully developed and erupted molars anchored to alveolar bone (AB): dental pulp (DPu) and periodontal ligament (PDL). (**E**) Legend for the cells depicted in A–D: dental pulp stem cell (DPSC) and bone marrow stromal cell (BMSC). This figure was created with BioRender.com (accessed on 16 November 2022).

Before examining the available research investigating craniofacial defects, it is important to understand the presence and structure of primary cilia in relevant cell populations. Primary cilia participate in every stage of tooth development and project from various dental cells in mice and humans, both *in vivo* and *in vitro* [5,17,18,19]. In addition to bone, cartilage, and fibrous tissue cells, primary cilia have been identified in cells of the dental epithelium, mesenchyme, lamina, and stalk, as well as in the following craniofacial cells: neural crest, periodontal ligament, dental pulp stem cells (DPSCs), odontoblasts, and ameloblasts (Figure 2) [7,9,13,18,19,20,21,22,23]. 

In some skeletal tissues, such as the growth plate, primary cilia are intentionally oriented to facilitate signaling interactions. Indeed, primary cilia in odontoblasts project into the dental pulp parallel to the dentin wall and ameloblast primary cilia emanate toward the outer enamel epithelium [19,24,25]. The intentional alignment facing away from mineralized tissue surfaces raises speculation that odontoblast and ameloblast primary cilia regulate cell polarity and matrix deposition [5]. A lack of primary cilia orientation has been reported in both neonatal and adult periodontal ligaments and the dental follicle [24]. 

In skeletal and kidney cells, the ciliary axoneme can adjust its length to tune primary cilia sensitivity. This could explain why primary cilium incidence and length were found to increase during DPSC odontogenic differentiation *in vitro*, as well as account for general observations that primary cilia are longer in regions with active cell differentiation and matrix production [7,23,26]. Primary cilia are also noticeably longer in the enamel knot, an established signaling center during tooth development [7]. Dental stalk cell primary cilia become very short or absent during keratinization in the tooth eruption process, which is consistent with observations in hair and skin [7,27,28]. 

Building evidence suggests that primary cilia operate as signaling hubs in various dental cells. The Hedgehog (Hh) and Wnt signaling pathways have been prioritized since their roles in craniofacial development are well-established and the primary cilium is known to coordinate these pathways in other skeletal cells. In this review, I focus on signaling in the dental epithelium and mesenchyme and neural crest cells, odontoblasts, and DPSCs because this information is available in detail. It is important to note that preliminary studies implicate a role for ciliary Hh signaling in periodontal ligament stem cells and this pathway has been speculated in other dental cells that will not be covered in this review [22].

## 4. Primary Cilia in Craniofacial Development

Considering primary cilia are prevalent in every craniofacial tissue, it is not surprising that disrupting this organelle has disastrous consequences in craniofacial structures. The vast majority of craniofacial primary cilia research has been conducted in the context of development or early postnatal growth. This is logical given that most craniofacial defects manifest neonatally, but the lack of investigation in standard maintenance, repair, and aging may also be indicative of technical limitations. Since primary cilium function can be disrupted by altering various components of the ciliary complex, I will examine craniofacial phenotypes resulting from different genetic mutations in this section. The symptoms associated with each phenotype are also summarized in Table 1.

### 4.1. Kinesin-like Protein Subunit 3a (KIF3A)

*Wnt1Cre;Kif3a^fl/fl^* mice were first generated to determine whether disrupting the primary cilium in neural crest cells could serve as a model for FND [9]. Indeed, mutant mice share a number of features observed in FND patients including cleft palate, bifid nasal septum, cranium occultum, and agenesis of the corpus callosum. Mutant embryos also lacked tongues, had mandibles that were wider and 30% shorter, and exhibited wider faces that became more exacerbated with time [9,29]. The facial widening resulted from drastically enhanced cell proliferation and upregulated Hh signaling [9]. More specifically, the authors noticed Hh signaling domains were expanded and speculated this increased the number of cells involved. *Wnt1Cre;Kif3a^fl/fl^* mice die at birth so tooth buds were implanted in renal capsules to evaluate dentin and enamel production [32]. Implanted buds gave rise to odontoblasts that were able to secrete matrix, but significantly less dentin was produced in mutant samples compared to controls. Mutant tooth buds were essentially devoid of enamel. Mutant enamel organs were found to be very disorganized with superfluous attempts at invagination, and the authors concluded this lack of structure combined with enhanced cell proliferation resulted in poor enamel deposition.

Additional studies were conducted to elucidate specific signaling mechanisms contributing to FND [29,32]. Motivated by reports of abnormal signaling in the dental mesenchyme from the FND phenotyping study, reporter mice were used to visualize regions with active Hh and Wnt signaling [9]. The dental mesenchyme was devoid of primary cilia but the epithelium was unaffected in *Wnt1Cre;Kif3a^fl/fl^* mutants. The loss of primary cilia resulted in decreased Hh signaling and increased Wnt signaling in the dental mesenchyme without changes in signaling domains in the epithelium [29,32]. However, these alterations interrupted signaling between the mesenchyme and epithelium. Following cues from the ectoderm, the dental mesenchyme signals to the epithelium to initiate formation of the cervical loops and enamel knots [33]. The authors therefore concluded that dental mesenchyme primary cilia are required to sense stimulation from the ectoderm and subsequently signal to the dental epithelium [29,32]. Moreover, the lack of response from the dental mesenchyme causes upregulated Hh signaling and downregulated Wnt signaling in the ectoderm [29]. Overall, primary cilia dysfunction gave rise to imbalances in Hh and Wnt signaling that entirely or partially disturbed morphogenesis of nearly every craniofacial structure. This work speaks to the primary cilium’s role as a signaling nexus capable of mediating multiple pathways within the same cell at the same time.

### 4.2. Intraflagellar Transport Protein 88 (IFT88)

Removing IFT proteins has proven to be a more specific mechanism for disrupting primary cilia since *Kif3a* was found to have non-ciliary effects on Wnt signaling in some tissues [34,35]. Genetic deletion of *Ift88*, a subunit of the IFT-B complex orchestrating anterograde transport from the ciliary base to the distal tip of the axoneme, is a popular tool for reducing primary cilium incidence and inhibiting its function. Neural crest primary cilia have also been disrupted using a *Wnt1Cre;Ift88^fl/fl^* knockout model [20,25]. IFT88 was selected as a candidate gene following genetic screening of a family with cleft lip and palate [25]. These mice die at birth due to several abnormalities including cleft lip and palate, tongue agenesis, and interrupted or improper formation of several structures in the face [25]. Specifically, mutant mice have wider facial midlines, indistinguishable palatine processes, smaller and misshapen mandibles, and missing incisors. This phenotype begins at E12.5 after neural crest cells have migrated into the facial prominences, suggesting primary cilium dysfunction does not affect cell migration or viability. Further investigation revealed cell proliferation was drastically reduced coinciding with fewer and shorter cilia in mutants, but cell migration and viability were unaffected *in vivo*. The authors also observed downregulated Hh signaling and upregulated Wnt signaling specifically in the dental mesenchyme, which is consistent with observations in the aforementioned *Wnt1Cre;Kif3a^fl/fl^* mouse model. Interestingly, another group identified expanded Hh signaling where an extra molar was formed mesial to the first molar in *Wnt1Cre;Ift88^fl/fl^* and Oak Ridge Polycystic Kidney mice, which contain a hypomorphic mutation of *Ift88* [20,36]. These contrasting findings are likely not contradictory, but illustrate the existence of distinct developmental niches and how primary cilia coordinate signaling according to the needs of the niche.

The role of primary cilia in enamel production has also been evaluated using a conditional deletion of *Ift88* in epithelial cells (*Keratin14;Ift88^fl/fl^*) [30]. *K14;Ift88^fl/fl^* mice had chalky incisors and roughened enamel surfaces on all molars due to abrasions that became more severe with time. The rough, chalky appearance resulted from thinner enamel layers that were less mineralized compared to control mice. Without functional cilia, enamel rod patterning was disorganized and fewer ameloblasts were present at the secretory stage, indicating primary cilia are important for enamel production. Signaling pathways known to be important for enamel formation—Hh, Wnt, FGF, and BMP—were evaluated but only Hh signaling was downregulated. Mutant mice treated with a Hh agonist demonstrated partial rescue of enamel thickness, indicating functional primary cilia are required for complete Hh signaling. *K14;Smo^fl/fl^* mice lacking a core component of the Hh signaling pathway were then generated to better examine the extent to which primary cilia mediate Hh signaling. The phenotype in these mutants, which did not survive birth, was much more severe compared to *K14;Ift88^fl/fl^* mice. This indicates Hh signaling operates independently of the primary cilium in certain developmental stages. The phenotypic disparity, combined with the observation that agonizing Hh signaling partially recovered enamel production, is evidence that the primary cilium may serve as a Hh signaling amplifier in this context. 

### 4.3. Intraflagellar Transport Protein 80 (IFT80)

IFT80 is another component of the IFT-B complex. Removal of IFT80 renders the primary cilium dysfunctional because key proteins are not trafficked along the axoneme to maintain its structure. Fibroblast growth factor 2 (FGF2) promotes proliferation, migration, and differentiation in dental pulp cells. One group hypothesized that FGF2-mediated FGF signaling is similarly important in odontoblasts and could be regulated by primary cilia. To test their hypothesis, they generated an *OsxCre;Ift80^fl/fl^* mouse model to disrupt primary cilia function in odontoblasts and dental pulp cells differentiating into odontoblast-like cells [23]. Primary cilium incidence was decreased in both odontoblasts and dental pulp cells, and odontoblast cilia were shorter in mutant pups *in vivo*. Mutant incisors erupted much later than controls during postnatal growth and later became malocclusioned. Molar eruption and crown formation appeared normal, but mutant roots were noticeably shorter. Cervical loop and odontoblast layers were thinner due to compromised cell proliferation and odontogenesis. Without functional cilia, odontoblasts lacked polarity and did not assemble into their usual alignment. Consequently, dentinal tubules were disorganized and secreted less calcified matrix. Mutants also exhibited decreased bone mass in the calvaria and alveolar bone, which is not surprising since *Osx* is expressed in osteoblasts. To study signaling mechanisms underlying the *OsxCre;Ift80^fl/fl^* phenotype, incisor dental pulp cells were isolated from these mice and primary cilia were disrupted using an adenoviral Cre to knockout IFT80. Hh signaling was reduced in knockout cells and decreased proliferation was found to be caused by attenuated FGF2–FGFR signaling. Interestingly, dental pulp cell primary cilia length was reduced with the knockout *in vitro*, suggesting the niche and/or mechanism of disruption can influence primary cilia length in certain cells. 

These results were recapitulated in DPSCs isolated from *Ift80^fl/fl^* mice and treated with adenoviral Cre [21]. Primary cilium incidence and length were reduced in knockout cells, coinciding with a significant reduction in odontogenesis. Expression of FGF receptor 1 (FGFR1) was markedly reduced in DPSCs lacking primary cilia, so they further investigated FGF signaling. FGF2 treatment enhanced odontogenic differentiation and enhanced cilium length in control DPSCs, but this behavior was lost in knockout cells. Hh and bone morphogenetic protein (BMP) signaling were also upregulated with FGF2 treatment in control cells, implying collaboration between Hh, BMP, and FGF signaling pathways. Indeed, agonizing Hh signaling lengthened cilia and enhanced the response in control cells, but neither this nor overexpressing BMP2 fully rescued the primary cilium deficit. This finding implicates the primary cilium as a signaling nexus that coordinates multiple signaling pathways to initiate proliferation, odontogenic differentiation, and subsequent mineralized dentin production in teeth. 

### 4.4. Intraflagellar Transport Protein 140 (IFT140)

IFT140 is part of the IFT-A complex, which directs retrograde transport from the axoneme tip to the ciliary base. An *OsxCre;Ift140^fl/fl^* model was generated to investigate the role of primary cilia in dentinogenesis due to IFT140′s direct connection to Joubert syndrome, Meckel syndrome, and CED [31]. At two and six weeks of age, mutant molars had comparatively shorter roots, thinner dentin layers, lower mineralization rates, and a significant reduction in dentin matrix protein production. Cementum-related lingual dentin was also thinner in mutant incisors, further suggesting odontogenesis was severely impaired. Incisor dental pulp cells isolated from these mice demonstrated reduced proliferation, odontogenic differentiation, and Hh signaling compared to control cells. These results are consistent with the findings in *OsxCre;Ift80^fl/fl^* mice, where a component of the IFT complex B was removed. The phenotypic overlap suggests primary cilium function is the driving factor in these disease states, rather than mutation of specific IFTs.

### 4.5. Other Primary Ciliary Genes Associated with Craniofacial Abnormalities

The aforementioned genes (KIF3A, IFT88, IFT80, and IFT140) were highlighted primarily because they are utilized in recent mouse models to study specific mechanisms underlying primary cilia dysfunction in craniofacial development. However, there are a wealth of other ciliary proteins linked to craniofacial abnormalities including, but not limited to, intraflagellar transport protein 20 (IFT20), centrosomal protein 290 (CEP290), Meckel syndrome type 1 (MKS1), oral–facial–digital syndrome 1 (OFD1), and ADP-ribosylation factor-like protein 13B (ARL13B). IFT20 is another IFT-B complex component and has been shown to be critical for collagen synthesis that facilitates craniofacial bone formation [37]. *Wnt1Cre;Ift20^fl/fl^* mice die shortly after birth due to breathing and feeding complications resulting from missing or malformed craniofacial structures [38]. This phenotype closely mimics those observed in *Wnt1Cre;Kif3a^fl/fl^* and *Wnt1Cre;Ift88^fl/fl^* mice, but the exact role in primary cilia function is complicated by IFT20′s non-ciliary role in collagen synthesis. 

**Figure 3:**
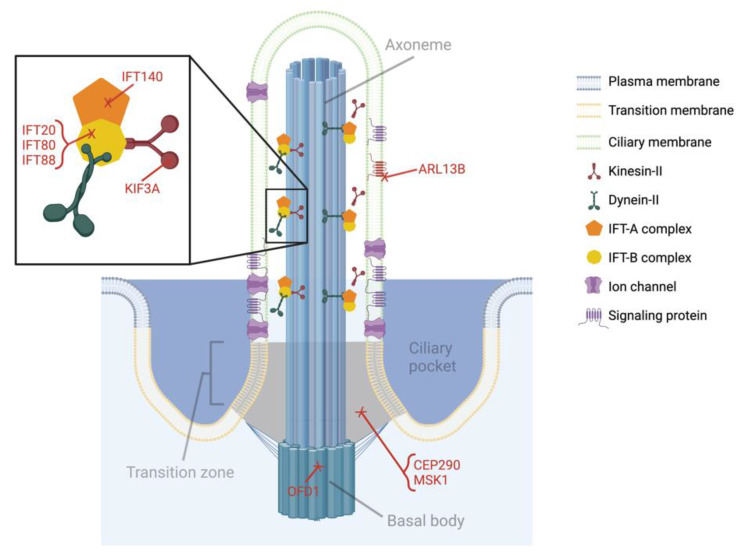
Primary cilium components associated with proteins linked to craniofacial abnormalities. Primary cilia function can be disrupted by altering a variety of components within the ciliary complex. Loss of KIF3A renders the motor protein kinesin-II non-functional. Removal of IFT proteins such as IFT20, 80, 88, and 140 alters the activity of IFT-A and IFT-B complexes, as well as their ability to bind motor proteins. Protein trafficking and localization are disrupted with mutations in proteins located in the ciliary membrane (ARL13B) or the transition zone (CEP20 and MSK1). The axoneme is unable to form normally when the basal body is destabilized from loss of proteins such as OFD1. This figure was created with BioRender.com (accessed on 14 November 2022).

In contrast to the IFT proteins, CEP290, MKS1, OFD1, and ARL13B are examples of how primary cilia function is compromised when components of the ciliary complex other than IFT are disrupted (Figure 3). Bardet–Biedl syndrome (BBS) was initially classified as arising from mutations in any of 14 ciliary genes, which became known as BBS proteins. A subset of the original BBSs form the BBSome, a complex that participates in protein trafficking and IFT. More proteins have since been classified as BBSs, including CEP290 and MKS1, which are linked to Joubert and Mekel syndromes. CEP290 and MKS1 are typically found in the transition zone and believed to be important for ciliary protein trafficking, but their exact regulatory mechanisms and role in craniofacial cells are unknown. OFD1 is named after the rare disease its mutation causes and is difficult to study in mice since the mutation is embryonically lethal in males and females die at birth from severe craniofacial abnormalities [39]. Furthermore, the mouse phenotype is more severe than what is presented in humans. In the absence of OFD1, the basal body is not positioned correctly and the axoneme fails to extend without proper anchoring [40]. This explains why primary cilia are essentially undetectable in knockout mice and the skeletal consequences are so severe [39]. ARL13B, a protein enriched in the ciliary membrane, is also implicated in Joubert syndrome yet remains unexplored in craniofacial development [41]. ARL13B is known for its roles in ciliary protein trafficking and regulating composition of the ciliary membrane, but building evidence suggests it may be an important link between the primary cilium and vertebrate Hh signaling [41]. If this is true for neural crest cells, elucidating the mechanisms of ARL13B could significantly advance our understanding of ciliary Hh signaling in craniofacial development.

## 5. Primary Cilia in Dental Repair

Compared to its investigation in craniofacial development, the role of the primary cilium in dental repair is largely unexplored. One study investigated primary cilia in tooth socket healing using the aforementioned *OsxCre;Ift140^fl/fl^* mouse model [42]. *Ift140* was targeted due to its high level of expression in the early stages of healing. Following tooth extraction, primary cilia were prevalent in cells within the defect site and the cilium incidence gradually decreased over time with healing. Mutant mice exhibited significant reductions in bone volume and expression of osteogenic genes compared to control mice, suggesting an impaired healing response. Alveolar bone marrow stromal cells (BMSCs) isolated from these mice demonstrated decreased cell viability, proliferation, osteogenesis, and mineral deposition in addition to reduced cilium incidence compared to control cells. Osteoclast activity did not appear to change, indicating the diminished socket healing was due to impaired osteoblast and BMSC activity. 

Another group interrogated the role of primary cilia in reparative dentinogenesis using the *OsxCre;Ift140^fl/fl^* model [31]. A shallow cavity was drilled into the first molars of these mice to encourage reparative dentin formation by lining odontoblasts, which express *Osx*. Dentin formation in the injury site and dentin matrix protein expression were significantly reduced in mutants. The authors interestingly noted that primary cilia were difficult to visualize near the injury site without treating the cavity. When treated, primary cilia were prevalent in cells near the exposure site in control mice, but cilium incidence was markedly reduced in mutants. This work complements a recent study showing Hh signaling is upregulated and *Gli1*-expressing dental pulp cells localized near the injury site in a similar dentin exposure model [43]. Considering primary cilia are well-established mediators of long bone repair and maintenance, it is important to further interrogate the primary cilium’s role in craniofacial bone and teeth repair [44,45,46,47].

## 6. The Future of Primary Cilia Research in Craniofacial Tissues

The innate healing potential of human teeth is very limited largely due to the slow rate of repair and lack of enamel regeneration. Primary cilia are important for mineralized tissue regeneration in bone and growing evidence suggests dental cell primary cilia have functions similar to those found in better-studied cells. Further investigation of this sensory organelle is required to better understand how dental cells can be manipulated, especially considering that primary cilia can be sensitized. For example, fenoldopam, an approved blood pressure medication, has been shown to lengthen osteocyte primary cilia such that osteogenesis is enhanced *in vitro* and *in vivo* [48,49]. Overexpressing ciliary proteins is also believed to amplify signaling coordinated by the primary cilium [50,51]. This section highlights future areas of exploration that will help elucidate the function and utility of primary cilia in dental cell behavior and accompanying development, maintenance, and repair of craniofacial tissues.

### 6.1. Mechanotransduction

Thus far, I have examined the chemosensory role of the primary cilium, but this organelle is also critical for sensing and converting physical external stimuli into intracellular signaling cascades, a process termed *mechanotransduction*. The primary cilium is a well-established mechanosensor in kidney and bone cells, and mechanosensory roles have been preliminarily demonstrated or speculated in other tissues [52,53,54]. Ciliary mechanotransduction occurs when an external stimulus causes the axoneme to bend, which subsequently activates stretch-activated proteins typically located near the ciliary base where the force is greatest in magnitude. The tooth experiences routine physical forces during eating and other physical stimuli can be introduced with dental work or injuries. The periodontal ligament displaces forces exerted on the tooth, providing a cushioned barrier between the tooth and alveolar bone. Long bone periosteal cells, which reside in a matrix similar to the periodontal ligament, require primary cilia to sense physical loading as the periosteum deforms and cells subsequently proliferate, differentiate, and deposit matrix as the force profile demands [55,56]. A similar role is possible for periodontal ligament cells during mastication and tooth movement. Primary cilia are randomly orientated in the ligament, providing further evidence of a mechanosensory function since the axonemes are positioned to sense physical movement from all directions.

Dental pulp cells and odontoblasts are also thought to be mechanosensitive. Pulp cells extracted from human exfoliated deciduous teeth (SHED) demonstrated enhanced mineralization and lengthening of the primary cilium when exposed to hydrostatic pressure [57]. When primary cilia were removed using chloral hydrate, mineralization was inhibited. Although chloral hydrate is not a preferred method for primary cilia disruption now that more specific and effective tools are available, this result is not surprising and dental pulp cell mechanosensation warrants further exploration [58]. Odontoblasts are hypothesized to sense dentin deformations and fluid flow, especially considering their long projections into the dentin [18,19,31]. Primary cilia in various cells are important for sensing matrix deformations, and osteocyte and kidney cell primary cilia are critical to sense and respond to fluid shear [52,54]. Given that odontoblasts secrete reparative dentin and primary cilia lengthen during odontogenesis, understanding the sensory role of odontoblast primary cilia could inspire new treatments to protect the dentin wall or even repair teeth.

### 6.2. Calcium Signaling

Calcium signaling is a well-established mechanism that triggers a variety of intracellular signaling cascades that promote tissue formation and maintenance. The primary cilium has been shown to mediate intracellular calcium signaling responses distinct from those that occur at the cellular membrane [59]. Mechanosensitive calcium ion channels are located within the ciliary complex to amplify signaling. In kidney epithelial cells, polycystins 1 and 2 (PC1/2) coordinate calcium signaling stimulated by the bending of the primary cilium axoneme in response to fluid shear. It was discovered that PC1 and PC2 are highly expressed in the primary cilium and functional cilia are required to transduce shear-mediated intracellular calcium signaling [52]. The PC1/PC2 complex is also present in odontoblast primary cilia, suggesting a similar mechanism may exist for calcium signaling in odontoblast mechanosensation [19,60]. Neuronal voltage-gated N-type (Cav2.2) calcium channels have also been found in odontoblasts and dental pulp cells [19,61]. In odontoblasts, Cav2.2 channels are clustered at the ciliary base and speculated to participate in stretch-activated intracellular calcium signaling [19]. Piezo calcium channels are highly expressed in SHED cells and their primary cilia lengthen when Piezo activity is agonized *in vitro*, resulting in enhanced odontogenesis [57]. Closer investigation of odontoblast primary cilia is especially justified considering these cells are chemo- and mechanosensitive, and there is strong evidence to suggest primary cilia are required to sense and respond to changes in the dentin microenvironment and for odontogenesis to occur. The polarity of odontoblast primary cilia further implies these cells are poised for dentin matrix deposition, which could be triggered by intracellular calcium signaling [31]. Elucidating calcium dynamics and their relationship to primary cilia function in odontoblasts could enhance our understanding of dentin production and repair to inspire novel therapeutics for dentin dysplasia and dental caries.

### 6.3. Innervation and Pain

Primary cilia have been implicated in neuronal migration, patterning, and axon guidance during development [12]. Nerve fibers can be intimately associated with the ciliary complex and the involvement of ciliary genes in diseases with strong neural phenotypes further suggests a role in axon extension and guidance [19,62]. In a recent study of Joubert syndrome, primary cilia were disrupted in projection neurons in mice using a *NexCre;Arl13b^fl/fl^* model [62]. Tissues were poorly innervated in mutants due to stunted and disorganized axonal projections, without changes in neuron viability. Dendrite outgrowth was significantly reduced and microfluidic experiments revealed that axon–axon signaling was impaired when primary cilia were dysfunctional. Direct stimulation and inhibition of neural primary cilia using photosensitive tools *in vitro* encouraged or prevented axonal growth, respectively, indicating the primary cilium rapidly controls neural growth. Axon projections are critical for neural signaling that facilitates tissue formation and maintenance, as well as pain. This study reveals an important role for neural primary cilia in craniofacial tissue development, but an important future direction is to determine the extent to which primary cilia also direct intercellular signaling in neural networks. It is speculated that the primary cilium does mediate signal transduction in neurons, but how this process perhaps regulates dental cell behavior and pain remains unknown [63].

Teeth are densely innervated to facilitate dental pulp viability and injury response. Sensory nerves secrete factors that promote mineralized tissue formation for standard tooth maintenance or in response to injury [43,64]. It is tempting to stimulate sensory nerves in favor of tissue production, but activating these nerves in patients could simultaneously elicit intense pain. As a signaling nexus and amplifier, the primary cilium is perhaps best equipped to balance tissue integrity with pain management. Targeted therapeutics that stimulate signaling at the level of the cilium could promote deposition of mineralized tissue without activating pain signals. Furthermore, determining how primary cilia orchestrate signaling between neurons and other dental cells would significantly advance our understanding of pulp viability, reparative dentinogenesis, and pain associated with dental caries, traumatic injuries, or genetic abnormalities. 

### 6.4. The Primary Cilium as a Signaling Nexus

Another proposed topic for further exploration is determining if and how dental cell primary cilia mediate various signaling pathways. The cilium operates as a signaling hub for the cell due to the dense collection of signaling proteins located in the ciliary complex. This trait also enables the primary cilium to detect low-magnitude stimuli and initiate signaling cascades in response, qualifying it as signaling amplifier. I previously covered development studies showing that primary cilia tune Hh and Wnt signaling to direct communication between the dental mesenchyme and epithelium [9,29,32]. This is comparable to studies in the long bone showing that primary cilia coordinate signaling between zones in the growth plate [65,66]. Odontoblast primary cilia alignment is also mirrored in proliferating zone chondrocytes, which stack into columns as they transition through the growth plate. Primary cilia align parallel to the columns to facilitate stacking, possibly through Wnt/PCP signaling [67]. These chondrocytes lose their polarity when primary cilia are disrupted, a behavior also seen in odontoblasts [23,66]. Many similar parallels exist between long bone/kidney primary cilia work and preliminary dental studies, so it is logical to hypothesize that dental primary cilia operate as signaling hubs. Indeed, dental cells utilize several signaling pathways that are associated with the primary cilium in other tissues including, but not limited to, Hh, Wnt, TGFβ, BMP, PTHrP, FGF, PDGF, and Notch signaling [67,68]. There is a wealth of tools available (genetic animal models, molecular interference, antibodies, biosensors, etc.) to study these pathways when primary cilium function is inhibited or enhanced both *in vitro* and *in vivo*. The lack of primary cilia knowledge is therefore not a consequence of technical limitations, and significant advances could be made in dental cell signaling if more time and resources are allocated to interrogating primary cilia.

## 7. Conclusions

**Figure 4:**
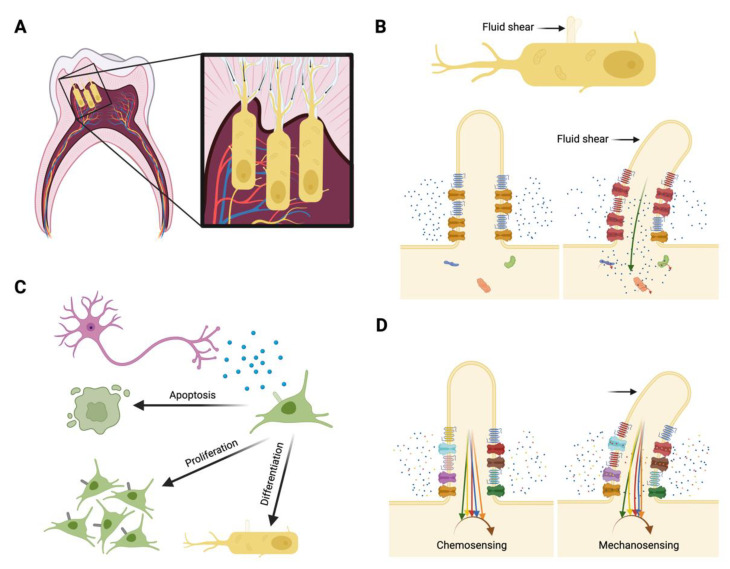
Summary of proposed future directions for dental primary cilia research. (**A**) Primary cilia may be important for odontoblasts (yellow) to sense physical stimuli from compression of the tooth and/or fluid shear (black arrows) through the dentinal tubules. (**B**) Fluid shear through the dentinal tubules may elicit an intracellular calcium response (green arrow) in odontoblasts that is mediated by the primary cilium. The axoneme bends in the direction of the fluid, causing calcium ion channels (yellow) to open (red) so that calcium (blue spheres) enters the ciliary complex and binds to proteins, triggering intracellular signaling cascades (red arrows). (**C**) During inflammation and injury, sensory nerves (purple) secrete factors that regulate dental stem cell (green) apoptosis, proliferation, and odontogenic differentiation in mechanisms that are potentially mediated by dental stem cell primary cilia. (**D**) Components associated with several signaling pathways (represented by different colors) are regulated simultaneously by the primary cilium in both chemo- and mechanotransduction. The individual contribution of each pathway is synchronized by the primary cilium to produce a coordinated intracellular response (brown arrow). This figure was created with BioRender.com (accessed on 15 November 2022).

The primary cilium is a unique sensory organelle that has been observed in nearly all craniofacial cells. It plays a key role in the development, maintenance, and repair of bones and teeth, and its dysfunction directly results in many craniofacial abnormalities. Although the genetic links between primary cilium function and craniofacial disease states are well established, very little is known regarding the role and behavior of dental cell primary cilia. In this review, I summarized existing information that demonstrates the importance of primary cilia in craniofacial research and motivates further investigation of this organelle. There is sufficient evidence to suggest primary cilia play a role in mineralized tissue formation, mechanotransduction, and dental and neural cell signaling, and these studies should be prioritized. In this review, I propose four future research directions of particular interest: (1) odontoblast fluid shear, (2) odontoblast intracellular calcium signaling dynamics, (3) signaling between nerves and dental cells, and (4) the simultaneous coordination of multiple signaling pathways in dental cells (Figure 4). This organelle is also an attractive therapeutic target since its sensitivity can be tuned to manipulate cell behavior distinct from other cytosolic processes. Overall, a better understanding of the primary cilium in craniofacial research will inform earlier treatment of developmental abnormalities, advance our knowledge of dental and neural cell behavior, and provide insight into tooth repair and pain management. 

## Figures and Tables

**Table 1:** Summary of reported craniofacial phenotypes in mouse models with disrupted primary cilia function.

Model	Mechanism of Disruption	Phenotype	Ref.
*Wnt1Cre;Kif3a^fl/fl^*	IFT motor protein impaired in neural crest cells	cleft palate, cleft skull, bifid nasal septum, cranium occultum, agenesis of the corpus callosum, missing tongue, shorter and wider mandibles, facial widening that is exacerbated over time	[9,29]
*Wnt1Cre;Ift88^fl/fl^*	IFT-B complex impaired in neural crest cells	cleft lip and palate, tongue agenesis, wider facial midlines, indistinguishable palatine processes, smaller and misshapen mandibles, missing incisors, extra molar	[20,25]
*Keratin14;Ift88^fl/fl^*	IFT-B complex impaired in epithelial cells	chalky incisors, roughened enamel surfaces on molars, thinner enamel layers in all teeth	[30]
*OsxCre;Ift80^fl/fl^*	IFT-B complex impaired in odontoblasts, osteoblasts, and differentiating DPSCs	Delayed incisor eruption, incisor malocclusion, short molar roots, thin dentin layers, decreased bone mass in calvaria and alveolar bone	[23]
*OsxCre;Ift140^fl/fl^*	IFT-A complex impaired in odontoblasts, osteoblasts, and differentiating DPSCs	Short molar roots, thin dentin layers, reduced mineralization rate and dentin matrix protein production	[31]
